# ORBITAL CELLULITIS IN PULMONARY TUBERCULOSIS: A CASE REPORT

**DOI:** 10.21010/ajid.v14i2.8

**Published:** 2020-07-31

**Authors:** Ni Made Inten Lestari, Susy Fatmariyanti, Hendrian D. Soebagjo, Neneng Dwi Kurniati, Delfitri Lutfi

**Affiliations:** 1 Ophthalmology Resident, Department of Ophthalmology, Faculty of Medicine, Universitas Airlangga/Dr. Soetomo General Academic Hospital, Surabaya, Indonesia; 2 Ophthalmologist, Orbital Oncology Division, Department of Ophthalmology, Faculty of Medicine, Universitas Airlangga/Dr. Soetomo General Academic Hospital, Surabaya, Indonesia; 3 Department of Medical Microbiology, Faculty of Medicine, Universitas Airlangga/Dr. Soetomo General Academic Hospital, Surabaya, Indonesia

**Keywords:** Extended spectrum β-lactamases, orbital cellulitis, pulmonary tuberculosis

## Abstract

**Background:**

Orbital cellulitis in immunocompetent patients with pulmonary tuberculosis (TB) is rare or unheard of. If left untreated, patients might lose their sight and potentially their life. This case describes orbital cellulitis due to extended spectrum β-lactamases (ESBLs)-producing bacteria in a patient with pulmonary tuberculosis.

**Materials and Methods:**

We report the case of a 47-year-old man referred to the emergency room in our hospital with swollen and painful right eye and face for 8 days. On admission, the patient condition was drowsy, pale, and feverish. Visual acuity of the right eye was only light perception with limited eye movement in all directions. The CT scan showed orbital sub-tissue swelling and sub-periosteal abscess on the upper lateral orbital wall. On day 5, pus culture was confirmed as extended spectrum β-lactamases (ESBLs) producing bacteria and Ziehl-Neelson staining test revealed acid fast bacillus with pulmonary inflammation highly pathognomonic of tuberculosis.

**Results:**

The patient showed significant clinical improvement on day 11. The patient was discharged on the day 15 in stable general condition with improved visual acuity on the right eye (capable of capturing hand movement).

**Conclusion:**

ESBL producing bacteria associated orbital cellulitis in tuberculosis patient potentially elevated the morbidity and possibly result in severe loss of visual acuity. Early diagnosis and treatment could reverse this comorbidity and produce a better outcome for affected patients.

## Introduction

Extended Spectrum β-lactamases (ESBL) orbital cellulitis in pulmonary tuberculosis is a relatively uncommon yet can occur (lee and Michael, 2011). In Indonesia, prevalence of tuberculosis is high and most commonly affects the lung but could also be extra pulmonary, manifestations of which can affect the eye and its surrounding tissues (Collins *et al.*, 2017). Orbital cellulitis potentially has a poor prognosis. If left untreated or therapy delayed, it is potentially sight and life threatening. According to reports, as many as 11% orbital cellulitis result in visual loss and intracranial complication (Chaudhry *et al.*, 2012). The standard management of acute orbital cellulitis is intravenous broad spectrum antibiotic along with treatment of associated symptom. When abscess is present, surgical drainage is necessary. To the best of our knowledge, this is the first case of orbital cellulitis and facial cellulitis due to ESBL producing bacteria in immunocompetent patient in pulmonary tuberculosis.

### Case Report

A 47-year-old man was referred to the emergency room in our hospital with swollen and painful right eye and face which had lasted 8 days. 3 days prior admission, the swelling seemed to be worsening and there was release of the pus.

The patient had a history of toothache since 2 years ago and bloody cough since 5 months ago. Further investigation did not revealed any history of trauma.

**Figure 1:**
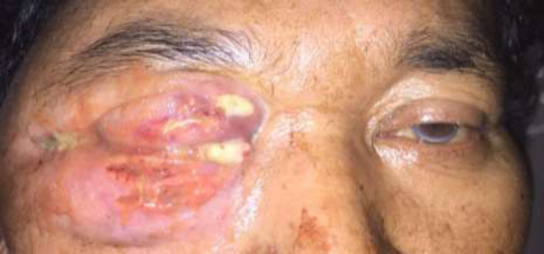
Non-axial proptosis, marked eyelid edema of upper and lower eyelid with purulent discharge

On admission, the patient presented with drowsiness, paleness, and fever. Visual acuity of the right eye was only light perception with limited eye movement in all directions. Examination revealed (Fig 1) non-axial proptosis, swelling of upper and lower eyelid with purulent discharge, chemosis, and purulent discharge on conjunctival bulbi and hazy cornea. There was fistula on right mandible to inner buccal which released pus.

**Figure 2:**
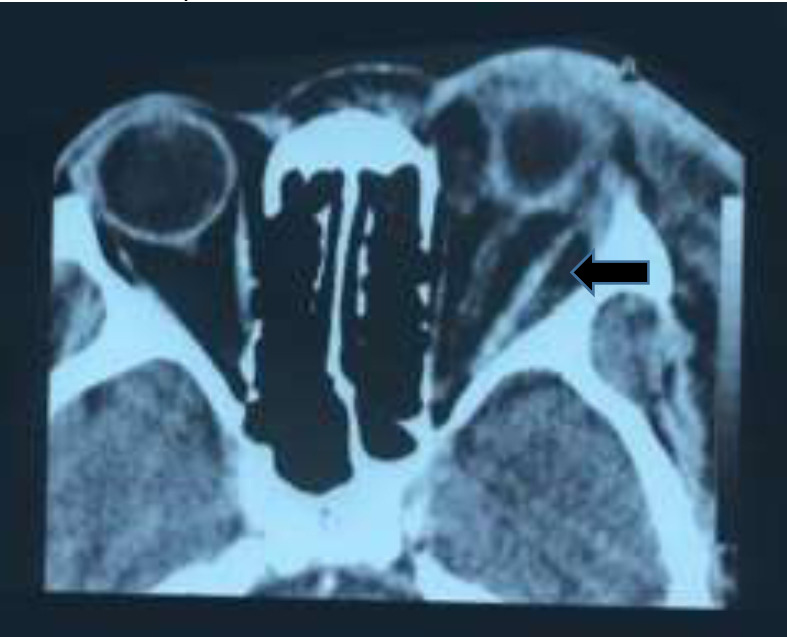
Axial view of CT scan showing orbital sub-tissue swelling and sub-periosteal abscess on the upper lateral orbital wall (black arrow).

Imaging modalities using CT scan (fig 2) revealed orbital sub-tissue swelling and sub-periosteal abscess on the upper lateral orbital wall. The laboratory test showed leukocytosis and elevated level erythrocyte sedimentation rate. A tentative diagnosis of orbital cellulitis with abscess formation was made. The patient was treated with intravenous ceftriaxone and metronidazole injection. The incision and drainage procedure was performed. The pus was collected, gram staining performed and culture done immediately.

**Figure 3:**
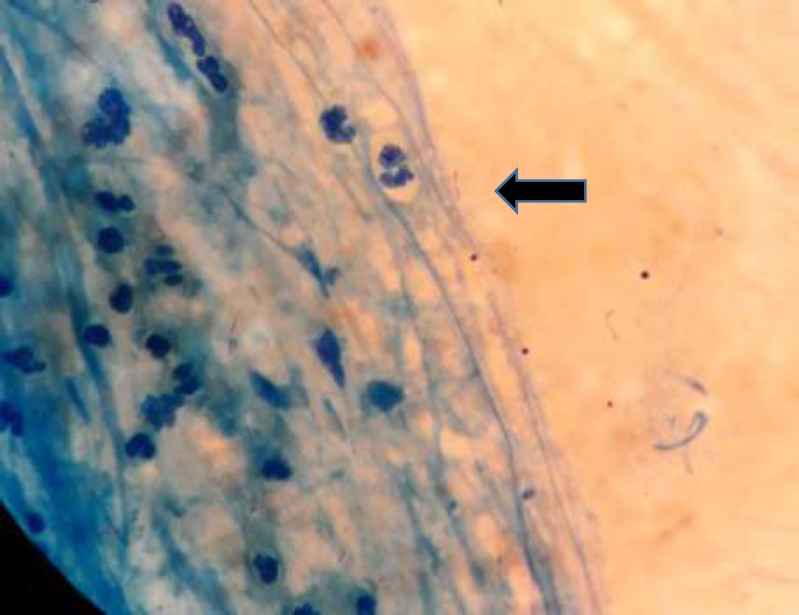
Ziehl-Neelson staining was found acid fast bacillus (black arrow)

We treated the patient conservatively in closed observation. On 3^rd^ day of treatment, patient still had fever, cough, swelling on the right eye and face with active pus production. We ordered sputum smear microscopy test and chest X-rays. On the 5^th^ day, the culture of the pus was confirmed using identification (ID) and antimicrobial susceptibility testing (AST), BD Phoenix (BD Diagnostic Systems, Sparks, MD) as extended spectrum β-lactamases (ESBLs) producing *Escherichia coli* and *Staphylococcus cohnii* ssp *urealyticum* with susceptibility to ampicilin-sulbactam. Ziehl-Neelson staining test (fig 3) revealed acid fast bacillus with pulmonary inflammation highly pathognomonic of tuberculosis. Based on the culture result, the antibiotic regimen was modified through a consultation with pulmonary division and patient was scheduled to receive fixed doses combinations (FDC) for tuberculosis. We managed patient with intravenous ampicillin sulbactam, intravenous metronidazole, with irrigation and drainage of the abscess once a day.

**Figure 4:**
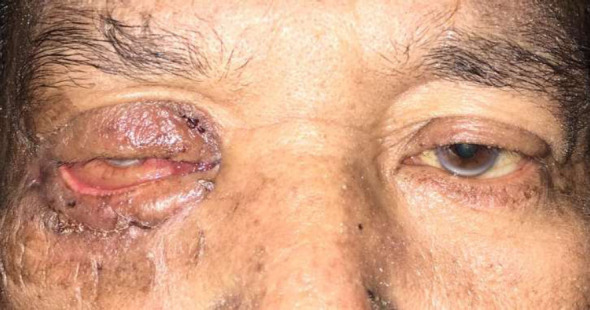
Day 15, Stable systemic condition with eyelid swollen and abscess resolved.

On the 11^th^ day, the swelling and abscess were resolved, followed by systemic condition improvement. The patient was discharged (fig 4) from the hospital on the 15^th^ day in stable systemic condition with visual acuity hand movement on the right eye.

## Discussion

Orbital cellulitis is infective process of the posterior tissue to the orbital septum. It originates from intraorbital sources, extension periorbital structures, exogenous, and endogenous causes (Akcay *et al.*, 2014; Rawat and Nair 2010).

The etiology of bacteria commonly reported from the abscesses of the orbit includes aerobes and anaerobes such as *Staphylococcus aureus, Staphylococcus epidermidis*, *Escherichia coli*, amongst others. ESBLs are β-lactamases capable of conferring bacterial resistance to the penicillins, cephalosporins; and aztreonam (but not the cephamycins or carbapenems).

Risk factors for infection include diabetes, geriatric patient, history of recent hospitalization and treatment with cephalosporins, penicillins and quinolones (Pereira *et al.*, 2010).

The best imaging to diagnose orbital cellulitis remains CT scan of the eye. It can reveal inflammatory stranding in the intraconal fat, increased density of intraconal or extraconal soft tissue mass, oedema of the extraocular muscle, intraorbital abscess, and sub-periosteal abscess (Sharma and Muzio, 2018). It is an urgent imaging indicated to assess the anatomy of the disease, evaluates sources of infection, cavernous sinus and intracranial involvement.

Empiric treatments with intravenous broad-spectrum antibiotics are immediately started once the diagnosis of orbital cellulitis is suspected until the result of the culture is confirmed. For infections where anaerobes (dental origin) might be expected, metronidazole is usually an option. Surgical drainage is indicated for significant underlying orbital, sub-periosteal abscess, and sinus disease. Urgent drainage (within 24 hours) is indicated where visual function is compromised, large orbital abscess is present, on evidence of intracranial extension, and known dental source infection (Lee and Yen, 2011; Dalvin, 2016).

In our case, the patient presented non-axial proptosis forward, ophthalmoplegia, chemosis on right eye, hazy cornea, and edema on maxillofacial region due to compressive effect of sub periosteal abscess, diffuse infiltration to orbital tissues and region maxillofacial dextra. On general examination, patient was drowsy, pale, and febrile probably due to sepsis. Surgical drainage contributes to the improvement of the condition by reducing proptosis and the administration sensitive antibiotic, also have significant effect in reducing the symptom. The presence of sub periosteal abscess contributes significantly to the optic nerve damage which could result in visual loss. Functionally, delayed therapy has poor prognosis.

Orbital cellulitis coexisting with pulmonary TB or ocular TB in immunocompetent patient is rare. A strong correlation between TB, poverty, and lack of education has been reported (Bidemi *et al.*, 2019: Narula *et al.*, 2010). In this case, patient has had 5 month-old hemoptoe which remained undiagnosed until hospitalization with orbital cellulitis. On Ziehl-Neelson staining test on his sputum, acid fast bacillus was found with inflammation leading to pulmonary TB (WHO, 2010). New case patient with pulmonary TB should have a regimen containing 6 months of rifampicin: 2HRZE/4(HR)3 receive a daily intensive phase followed by three times weekly continuation phase provided that each dose is directly observed (H = isoniazid, R = rifampicin, Z = pyrazinamide, E = ethambutol, S = streptomycin). On day 5, after diagnosed pulmonary TB without multi drug resistant (MDR) was established, patient received TB regimen as a new case patient (WHO, 2010; Shashank, 2012).

Improvement of the patient can be monitored with clinical condition (edema, erythema, reduced pus production), general status (normal vital sign), and confirmed by laboratory test (normal leucocyte, and inflammation marker). On day 1 after the therapy, the patient showed improvement and significant improvement clinically on day 11^th^. Regular follow up was done 1 week, 2 weeks, and 1 month to monitor the sequel sign.

## Conclusion

We reported a case of ESBL-bacteria associated orbital cellulitis in pulmonary tuberculosis presenting with swollen and painful right eye and face with decrease visual acuity. Early diagnosis and treatment could reverse this comorbidity and produce a better outcome for affected patients.

**List of Abbreviations:**
ESBLs: : extended spectrum β-lactamases
FDC: : fixed doses combinations
CT: : Computerized Tomography
TB: : Tuberculosis.
